# Feeding Problems in Young Children: A Cross-Sectional Study in Sweden

**DOI:** 10.1097/PG9.0000000000000297

**Published:** 2023-03-09

**Authors:** Kajsa Lamm, Kajsa Landgren, Runar Vilhjálmsson, Inger Kristensson Hallström

**Affiliations:** From the * Department of Health Sciences, Faculty of Medicine, Lund University, Sweden; † Faculty of Nursing, School of Health Sciences, University of Iceland, Iceland.

**Keywords:** feeding problems, prevalence, pediatric feeding disorder, BPFAS

## Abstract

To describe the prevalence of feeding problems (FPs) in children aged 10, 18, and 36 months who visited Swedish Child Health Services.

**Methods:**

Parents of children attending regular 10-, 18-, and 36-month visits at the child health care centers (CHCCs) in Sweden answered a questionnaire including a Swedish version of the Behavioral Pediatrics Feeding Assessment Scale (BPFAS) as well as demographic questions. CHCCs were stratified according to a sociodemographic index.

**Results:**

Parents of 238 girls (115) and boys (123) completed the questionnaire. Using international thresholds for FP detection, 8.4% of the children had a total frequency score (TFS) indicating FP. Based on the total problem score (TPS), the result was 9.3%. The mean score for all children was 62.7 for TFS (median 60; range 41–100), and 2.2 for TPS (median 0; range 0–22). Children aged 36 months had a significantly higher average TPS score than younger children, but TFS scores did not differ by age. There were no significant difference in gender, parents’ education, or sociodemographic index.

**Conclusion:**

Prevalence numbers found in this study are similar to those found in studies with BPFAS in other countries. Children 36 months of age had a significantly higher prevalence of FP than children aged 10 and 18 months. Young children with FP should be referred to health care specializing in FP and PFD. Creating awareness of FP and PFD in primary care facilities and child health services may facilitate early detection and intervention for children with FP.

**What Is Known**

- Feeding problems (FPs) can be understood as a continuum ranging from milder transient FP to severe feeding disorders, including pediatric feeding disorder.
- There is a lack of prevalence studies and studies regarding children under the age of 2.

**What Is New**

- Prevalence of FP in Sweden measured with Behavioral Pediatrics Feeding Assessment Scale (BPFAS) is 8.4%/9.3%.
- Mean scores on BPFAS are similar to other western countries.
- Children aged 36 months were more likely to have FP than those aged 10 or 18 months.

## INTRODUCTION

Feeding problems (FPs) in children can be understood as a spectrum, ranging from mild, transient difficulties, via picky or selective eating to severe behavioral and/or medical feeding disorders (FDs), including tube feeding dependency (1–4). The age period up until 2 years of age is critical in feeding development, and FP typically starts early, at 6 months to 4 years of age (2,5–7). FPs are common, but a plethora of definitions and classifications complicates comparing prevalence numbers (2,8).

A Swedish study from 1986 found a 1.4% prevalence of FP for infants aged 3–12 months (9), whereas other studies have found FP affecting 25%–45% of children in general populations (6,10,11), with 25% being an often-cited number (11). The disparity in definitions, historically organized in an organic/nonorganic dichotomy, paved way for the pediatric feeding disorder (PFD) consensus definition, which was introduced in 2019 (12) and received ICD-10 codes in the United States in 2021 (13). PFD is defined as an impaired oral intake not age-appropriate, lasting over two weeks and associated with dysfunction in one or more of 4 domains: medical, nutritional, feeding skill, and/or psychosocial (12). There is a lack of PFD prevalence studies using a population-based sample, as well as studies on younger children (2), and the prevalence of FP in Sweden is unknown. To support, develop, and provide care for children with FP, early detection of FP is vital. Therefore, the aim of this study was to describe the prevalence and degree of FP in typically developing children <3 years of age in Sweden.

## METHODS

This was a descriptive cross-sectional study, with a questionnaire distributed to parents at their child’s regular visits at the child health services (CHS). Primary outcome was prevalence of FP using internationally established cutoff levels (8,14). A secondary outcome was to explore frequency of FP with regards to age and socioeconomic variables.

The study was performed in CHS in the region Skåne, Sweden, with approximately 1.4 million inhabitants. The CHS are organized via child health care centers (CHCCs) and attended by almost 100% of children up to 6 years of age (15). CHCCs are publicly funded, and care is free of charge, irrespective of if they are publicly or privately run. A socioeconomic index, Care Need Index (CNI) is used to allocate public resources for primary care, in which the CHCCs are included. Higher rates of unemployment, single marital status, children <5 years of age, residential mobility, being born outside of Europe, and low educational status in the catchment area, renders a higher CNI and thus more public funding (16). Nurses provide the bulk of care and support for parents, and children receive health surveillance by child physicians at regular intervals. Care and support follow the national child health program (17), including national guidelines concerning food introduction (18). When needed, children can be referred to specialists such as child physicians, dieticians, psychologists and speech and language pathologists.

Inclusion criteria were parents >18 years of age, able to understand spoken and written Swedish, with a child attending the regular pre-booked health visits for ages 10, 18, and 36 months. Power analysis based on an estimated prevalence for FP of 25% (11), margin of error of 5%, and a 95% confidence interval determined that parents of 289 children were required for the analysis. Assuming that 30% of parents asked for participation would chose not to, we aimed to include 376 parents.

The questionnaire included (1) questions on parent’s age, education, and if the child had received help for feeding-related issues or other health-related issues, had any allergies, and if the child had siblings, (2) a Swedish version of the Behavioral Pediatric Feeding Assessment Scale (BPFAS) (14,19). BPFAS has been thoroughly tested for validity and reliability in different contexts (20–22), is the most frequently used parental questionnaire on children’s FP (2), and is reported to be reliable (23). Taking approximately 15 minutes to complete, BPFAS has 35 items. The first 25 involve frequency of different aspects of the child’s eating behaviors (like “my child eats fruits,” “my child chokes or gags at mealtimes”). The following ten items concern the parent’s reaction to the child’s behavior. Statements are phrased both positively and negatively, and answers are given on a 5-point Likert scale. Each item has a follow-up question where the parent is asked if the behavior is a problem for the parent or not, yes or no being the alternatives. Based on the sum of the 35 items, a total frequency score (TFS) is created ranging from 35 to 175, and a total problem score (TPS) is created summing up of the follow-up questions, ranging from 0 to 35. Thresholds for detecting clinically significant FPs are a TFS score of above 84, and a TPS score above 9 (8,14).

**FIGURE 1.**
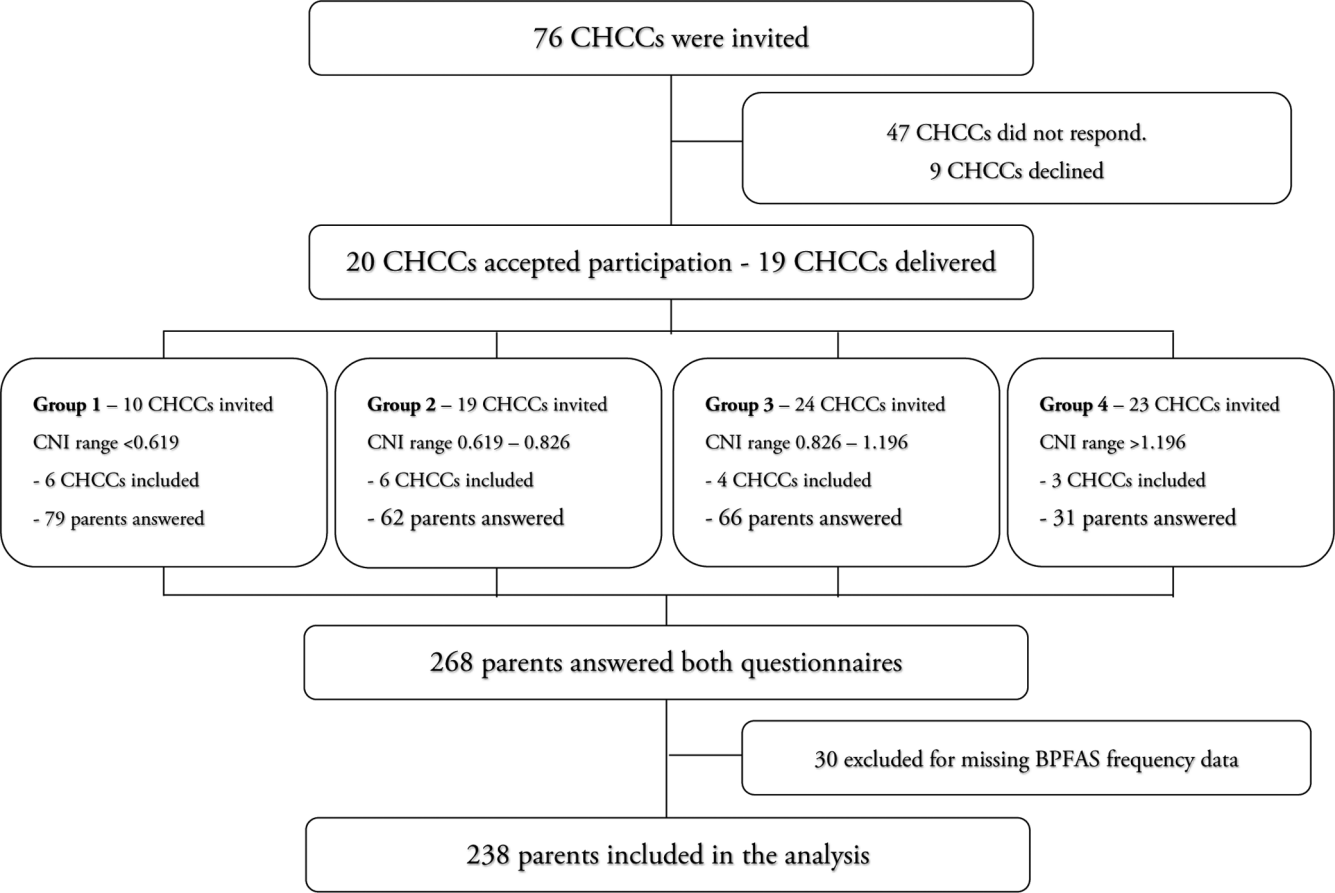
Flowchart of the included CHCCs and number of answers from parents. CHCCs = child health care centers.

The study was performed in the western part of Skåne, where the 101 CHCC’s had a range of CNI from 0.32 to 2.45 (mean = 1, median = 0.8). The CHCCs were collapsed into four CNI groups based on quartiles (Fig. 1) with CNI 1 being lowest and CNI 4 highest in terms of catchment area health care needs. To achieve a broad sociodemographic sample, the same number of CHCCs was drawn from each group. CHCC nurses were instructed to deliver questionnaires consecutively to parents of children who visited their regular 10-, 18-, and 36-month visits. Nurses informed parents about the study and if they wanted to participate, parents were given an envelope containing the questionnaire, and information about and instructions on how to complete the study. Questionnaires were distributed and collected from January 2020 to January 2022 and could be completed in 3 ways: (1) in paper form at the CHCC; (2) at home and returned in a prepaid envelope; or (3) answered online. The online option was offered via the web-based REDCap application (Vanderbilt REDCap, version 8.1.7) and could be reached via web link or a QR code. Of the included participants, 141 parents (59.2%) posted the questionnaire, and 97 (40.8%) answered online. Paper versions were entered into REDCap by the first author. Figure 1 describes the inclusion process of CHCCs and parents.

Numbers and percentages were calculated for TFS and TPS by children’s age and CNI groups. Of the 268 answers, 30 had missing data in the BPFAS frequency section and were excluded. Of the 238 included questionnaires, very few had missing demographic data: parent age (7), gender (2), siblings (1), and if the parents had sought healthcare for child’s FP (1). Chi-square tests were used to determine significance regarding differences in numbers of children with scores above thresholds for TFS and TPS. The Kruskal–Wallis H-test was used to determine if there was a significant relationship of TFS and TPS scores with the children’s age and gender, the CNI group of the CHCCs, and parent’s education. Data were exported from REDCAP to IBM SPSS Statistics version 28.

Ethical approval was obtained from the Swedish Ethical Review Authority (191105/2019-04577). Parents were informed about the study, that participation was voluntary and declining to participate would not change the care provided by the CHCC. All data were handled confidentially and only at the group level.

## RESULTS

A total of 238 questionnaires were included in the analysis, answered by parents aged 23–47 years (median 34). Background data for the parents and children (115 girls and 123 boys) are described in Table 1. Seventeen (7.1%) of the parents stated that they had had contact with health care personnel specifically regarding FP for their child (ie, had met a child physician, dietician, pediatric nurse including the CHCC nurse, speech and language pathologist, and/or psychologist). They furthermore reported that 14 (5.9%) of the children had food allergies (eggs, milk protein, avocado, banana, kiwi, or gluten intolerance). Parents of 13 (5.6%) of the children had been in contact with the health care system regarding illnesses including asthma, constipation, intermittent rash, tonsil problems, investigation for intermittent fever and infections, feet problems, length growth faltering, and a congenital syndrome.

**TABLE 1.** Background data of parents and children

	%	n
Parent—female	85.3	197
Parent education	0.8	2
Compulsory school	26.6	63
High school	67.9	161
Tertiary education	4.6	11
Other		
More than one child in the family	62.6	153
Contact with health care regarding illness	5.6	13
Contact with health care regarding FP	7.1	17
Children with food allergies	5.9	14

FP = feeding problem.

### Prevalence of FP

According to parent reports in BPFAS, 8.4% of the children (n = 20) had a TFS above 84, indicating FP. For TPS, 9.3% of the children (n = 22) had a score above 9, indicating FP. Divided into age groups, 4% of the children in the 10-month group (n = 3) scored above the threshold for TFS and TPS, respectively. In the 18-month group, 6% of the children (n = 5) were above threshold for TFS, and 7.2% (n = 6) were above threshold for TPS. In the 36-month group, 15% (n = 12) were above threshold for TFS, and 16.3% (n = 13) for TPS.

The difference between age groups was significant for TFS (Chi-square = 7.02, df = 2, *P* = 0.03) as well as for TPS (Chi-square = 7.43, df = 2, *P* = 0.024). Regarding gender, the result for TFS above 84 was 9.6% (n = 11) for girls and 7.3% (n = 9) for boys. For the TPS, 12.2% (n = 14) of the girls and 6.5% (n = 8) of the boys were above the threshold. The gender difference was not significant, and the same was true regarding the relationship between parent’s education, CNI group, and being above or below TFS or TPS thresholds.

### The Distribution of TFS and TPS Scores

The mean TFS score for all age groups was 62.7 (range = 41–100, median 60, SD = 12.4), and the mean TPS score was 2.2 (range = 0–22, median 0, SD = 4.6). Neither the CNI group, child’s gender, nor parent’s education was significantly associated with TFS or TPS scores. The TFS mean for girls (n = 115, 48.3%) was 63.8 (range = 43–100, median 61, SD = 12.6), and for boys (n = 123, 51.7%), it was 61.6 (range = 41–97, median 60, SD = 12.2). For TPS, the mean for girls was 2.7 (range = 0–21, median 0, SD = 5.2) and for boys 1.8 (range = 0–22, median 0, SD = 4). Regarding age group, the results differed between TFS and TPS scores. The 36-months-old children had a TFS mean score of 66.1 (median 63, SD = 14.5) and a TPS score of 3.7 (median 1, SD = 6). The latter was significantly higher than corresponding scores for the 10- and 18-month children (*P* < 0.001). For TFS, there was no significant difference between the age groups (*P* = 0.072) (see Table 2). The results for TFS and TPS scores were further divided by CNI and age groups are shown in Table 2.

**TABLE 2.** Results for TFS and TPS divided by age group and CNI group

CNI group	TFS 10 mo	TFS 18 mo	TFS 36 mo	TFS all ages	TPS 10 mo	TPS 18 mo	TPS 36 mo	TPS all ages
CNI 1								
Number	25	28	26	79	25	28	26	79
Mean/SD	58.2//9.4	60.4//13.2	65.2//14	61.3//12.6	0.9//2	2//4.8	3.9//6	2.3//4.7
Median	56	60	62.5	60	0	0	1.5	0
CNI 2								
Number	19	21	22	62	19	21	22	62
Mean/SD	61.7/10.4	59.2/9.4	70.1/17.6	63.4/13.9	1.4/4.4	1.5/3.2	4.9/6.9	2.7/5.3
Median	59	59	97	60.5	0	0	1.5	0
CNI 3								
Number	22	23	21	66	21	23	21	65
Mean/SD	63.4/12	61/11.3	64.2/14	62.9/12.3	1.3/2.8	1.6/4.2	3/6.3	2/4.6
Median	59.5	60	61	60	0	0	0	0
CNI 4								
Number	9	11	11	31	9	11	11	31
Mean/SD	64.8/8	62.5/8.7	63.6/9.5	63.6/8.6	1.3/2.6	1.3/1.7	2.3/3.4	1.6/2.6
Median	67	64	60	65	1	0	1	1
All groups								
Number	75	83	80	238	74	83	80	237
Mean/SD	61.4/10.4	60.6/11.2	66.1/14.5	62.7/12.4	1.2/3	1.7/3.9	3.7/6	2.2/4.6
Median	59	60	63	60	0	0	1	0

CNI = care need index; TFS = total frequency score; TPS = total problem score.

Of those 17 children whose parents stated having had contact with health care personnel regarding FP for their child, the mean for TFS was 74.3 (median 74, SD = 12.8) and for TPS it was 6.12 (median 4, SD = 6.2). Four of the 17 children were above the threshold for TFS, and 5 above for TPS.

## DISCUSSION

Our data show that 8.4%/9.3% of children in ages 10 months to 3 years may have FP serious enough to warrant a referral for further assessment and intervention.

Milano et al (24) suggest that around 10% of children may have FPs that require intervention, and prevalence numbers of FP in the present study are comparable with 8.2% in a study from Greece (8), even though the children in the present study are younger. For TPS, the results from our study (9.3%) are considerably lower, with the Greek study having a TPS result of 26.6%. Sdravou et al argued that the deviation in percentages of the two scores might reflect a differentiation in severity of the FP, and that TPS is more sensitive to “milder” FP, which would not seem to apply to Swedish children in the present study. Significantly more children in the 36-month group scored above the thresholds, possibly mirroring the assumption that parents allow younger children more scope when feeding, and children are expected to become more independent and competent feeders as they develop. Although the prevalence rates were lower than assumed, it is important that all children with potential FP and PFD with clinical significance are identified. Therefore, children should be referred for further evaluation when either of the BPFAS scores is above threshold.

The mean TFS for all children in the present study was 62.7, similar to TFS reported in Greece, 62.7 (8), and in the United Kingdom 62.5 (20), but slightly below the mean TPS reported in Canada, 63.9 (14). The mean TPS in the present study (2.2) was lower than reported in the United Kingdom (2.7) (20) and Canada (3.0) (14), possibly due to including younger children in the present study. The Greek study found a mean of 6.2 on TPS, but the authors of that study attribute this to cultural differences (8).

Children in the present study are younger than in the above mentioned earlier studies (8,14,20) with BPFAS, apart from the Canadian study (14). This might be reflected in the significant correlation of age and mean scores on TPS, and corroborates earlier findings that FP is less prevalent in infants and younger children. Earlier work has found similar results, for example, an increase in perceived pickiness among children, from 19% to 50% from 4 to 24 months (7).

In line with other studies (7,8,14), there was no significant differences regarding socioeconomic factors and FPs in the present study. This is important when allocating public resources. Furthermore, gender does not seem to be a significant factor when measuring with BPFAS, as found by both the present study and Dovey et al (20).

This is the first study using BPFAS with parents of children attending regular health visits in a Swedish setting and the first prevalence study in the country since 1986 (9), contributing to the knowledge of FP. Determining exact prevalence numbers for FP and PFD is difficult, due to inconsistency in terminology, study design and instruments used (21), but as Marshall et al (21) stated, also using BPFAS, parent-reported feeding profiles differ between children with FD and children without FD, both regarding the frequency of behaviors and what is considered a problem by the parents (21). Using medical registers, Kovacic et al (25) searched ICD codes to capture PFD in children aged 2 months up to 5 years, finding an annual prevalence of 2.7%–4.6% (25). These numbers were found in the United States where the health care system is quite different from the Swedish. The Swedish version of the BPFAS has not been tested for validity and reliability, which is a limitation of the study. Even so, the prevalence numbers found in the present study are similar to other studies using the BPFAS (8,14), and the prevalence detected in the present study does indicate that FP and PFD are not uncommon.

A strength of the study is that it targets an age group where more research is needed (2). Furthermore, it is not undermined by a selection effect since virtually all children are seen by the Swedish CHS and the CHCCs are stratified according to CNI. To control for selection bias by the CHCC nurses, they were instructed to recruit participants consecutively regardless of whether the nurse knew of any FP or not. Fewer CHCCs in CNI group 4 participated, and therefore, the number of participants from this group was lower. Due to COVID-19, it was not possible for the first author to recruit parents on site at the CHCC, which may have resulted in fewer participants. However, this did not undermine statistical power estimations since prevalence rates were lower than assumed in the power analysis. To ensure methodological rigor, the STROBE checklist was followed (26).

BPFAS was developed with the intent of capturing problems during mealtimes. It has been used as a screening tool to detect clinically relevant FP (21), and can be used in subpopulations such as children with neurological impairments and cancer (27,28). Even so, the use of BPFAS for detecting the prevalence of the new consensus definition PFD might be considered problematic, since BPFAS was constructed using older, more dichotomous criteria of organic or nonorganic FDs, intended to define if there is a problem during mealtimes. Nevertheless, BPFAS does capture the perceived frequency of difficulties with feeding skills and some nutritional aspects (child eats vegetables, carbohydrates, protein, and need of feeding tube), but may not cover all of the 4 domains in PFD: medical, nutritional, feeding skill, and psychosocial. The latter is probably accurately caught by BPFAS, but it may not catch the medical domain of PFD. There are items covering the frequency of choking, gaging, and vomiting, but these behaviors may not always be related to a medical dysfunction.

Typically, clinical observation, interview, and questionnaires are commonly used to assess FP in children (8), but in the CHS, the nurse must rely on growth charts and the dialog with the parents. Parents do, however, report that their concerns about their child’s feeding difficulties are not always listened to (29), and to ensure that children with FP and possible PFD get help early, a parental questionnaire may be useful. BPFAS is used for research purposes in different countries and populations, but this does not necessarily make it the most appropriate instrument when screening for FP and PFD. To use BPFAS as a tool in CHS and tertiary health care, national threshold scores for different ages should be derived, to adjust for cultural differences. BPFAS is complex to score, some of the items have room for interpretation and it might take too long time out of a health care visit compared to other screening tools. Therefore, a shorter instrument might be preferable for administration and assessment in clinical settings.

## CONCLUSIONS

Our findings suggest that prevalence rates of FP in Sweden reported by parents are similar to those in other western countries. Children 36 months of age had a significantly higher prevalence of FP than children aged 10 and 18 months, but FP was not associated with socioeconomic status or gender. As FP might progress to PFD, knowing the prevalence for FP and PFD is an important part in creating awareness in primary care facilities and in CHS, and facilitating earlier access to health care for parents and children. Young children with FP should be referred to health care specializing in FP and PFD.

## ACKNOWLEDGEMENTS

Thank you to Helena Johansson, Uppsala University, for the use of the Swedish version of BPFAS, and to Anna Åkesson for contributions regarding the statistical work.
